# Effects of Sensory-Enhanced Acute Exercise on Affective Characteristics of Employees

**DOI:** 10.3390/bs15020202

**Published:** 2025-02-13

**Authors:** Tanja Lazarević, Aleksandar Nedeljković, Stanimir Stojiljković, Ana Vesković, Saša Bubanj, Novica Bojanić, Aleksa Bubanj, Ana Orlić

**Affiliations:** 1 Faculty of Sport and Physical Education, University of Belgrade, 11000 Belgrade, Serbia; tanjalazarevic976@gmail.com (T.L.); aleksandar.nedeljkovic@fsfv.bg.ac.rs (A.N.); stanimir.stojiljkovic@fsfv.bg.ac.rs (S.S.); ana.veskovic@fsfv.bg.ac.rs (A.V.); ana.orlic@fsfv.bg.ac.rs (A.O.); 2 Faculty of Sport and Physical Education, University of Nis, 18000 Nis, Serbia; 3 Faculty of Medicine, University of Nis, 18000 Nis, Serbia; bojanicnovica@gmail.com (N.B.); bubanjaleksa@gmail.com (A.B.)

**Keywords:** anxiety, motivation, emotion, sensory modulation

## Abstract

Employee well-being and affective states are critical factors influencing overall organizational success. This study examined the immediate effects of a sensory-enhanced acute exercise program on employees’ affective characteristics, including emotions, anxiety, and work motivation; A total of 84 participants, split into an experimental and control group, participated in the actual study. The experimental group engaged in a 14-min tailored exercise program in a sensory-rich “smart room” while the control group watched a neutral animated documentary. A pretest–posttest design was used, and data were analyzed using repeated measures ANOVA with post hoc tests for significant interactions; The results revealed significant improvements in positive emotions (F(1, 82) = 20.99, *p* < 0.01) and work motivation (energy level: F(1, 82) = 48.36, *p* < 0.01; emotional arousal: F(1, 82) = 12.29, *p* < 0.01) in the experimental group, along with a significant reduction in anxiety (F(1, 82) = 11.37, *p* < 0.01) compared to the control group. Although reductions in negative emotions were observed across both groups, the differences were not statistically significant; This study underscores the effectiveness of integrating exercise with tailored sensory environments to enhance emotional states and workplace motivation. Such interventions offer a practical and scalable approach to improving employee well-being, highlighting their potential for adoption in diverse professional settings.

## 1. Introduction

Employee engagement is crucial in every profession as it directly influences the overall success and growth of an organization. High productivity leads to better use of time and resources, ensuring tasks are completed efficiently and effectively (Bakker, 2011). This, in turn, enhances the quality of output, whether it is products or services, allowing businesses to meet customer demands and maintain competitiveness in their respective industries (De Menezes & Kelliher, 2011). Additionally, when employees are more productive, organizations can reduce operational costs, as fewer resources are wasted and more value is generated from the workforce (Bloom et al., 2012). Effective engagement also boosts morale and job satisfaction, as employees who work efficiently often experience a sense of accomplishment (Bakker & Demerouti, 2008). Ultimately, the consistent engagement of employees contributes to the sustainability and profitability of the organization, no matter the industry (Bailey et al., 2017). From the perspective of Organizational Ecology Theory, productivity can also be seen as a result of an organization’s ability to adapt to internal and external environmental pressures, making employee well-being and effective interventions essential to the overall health and resilience of the organization (Fuertes-Callén et al., 2022).

Employee engagement is closely intertwined with their affective characteristics, including positive and negative emotions, anxiety, and current motivation levels. Positive emotions, such as enthusiasm and satisfaction, have been shown to boost work performance and creativity while simultaneously reducing stress and enhancing teamwork (Fredrickson, 2013; Bakker & Demerouti, 2017). In contrast, negative emotions related to stress or frustration can narrow attention, decrease work efficiency, and increase the likelihood of errors (Schaufeli, 2015). Workplace anxiety often leads to reduced engagement by impairing concentration and placing greater demands on cognitive resources, ultimately slowing decision-making and diminishing creativity (Lepine et al., 2005). Moreover, the degree of an employee’s motivation significantly impacts engagement. Highly motivated employees exhibit greater perseverance, focus, and willingness to tackle challenges, whereas low motivation is associated with procrastination and reduced work output (Ryan & Deci, 2000; Gagné & Deci, 2005). Empirical evidence suggests that affective states can fluctuate throughout the workday, with interventions aimed at promoting positive emotions and mitigating stress, which have a direct impact on performance (Fisher, 2010; Sonnentag, 2015). Consequently, understanding and managing the affective characteristics of employees represent a strategic priority for enhancing engagement within modern workplaces (Cropanzano et al., 2017).

Physical exercise, as a modifiable independent variable, plays a crucial role in shaping the affective characteristics of employees, significantly impacting their emotions, anxiety levels, and overall motivation. Studies consistently demonstrate that engaging in physical activity can lead to enhanced positive emotions, such as increased vigor, contentment, and reduced perceptions of stress, thereby improving work-related mood and engagement (Rebar et al., 2015; Ekkekakis, 2017). Acute bouts of exercise, such as a single session of aerobic activity, have been shown to elevate mood and reduce tension and anxiety immediately following exercise (Basso & Suzuki, 2017). Moreover, regular physical activity contributes to sustained reductions in anxiety levels by modulating stress responses and enhancing emotional resilience (Anderson & Shivakumar, 2013). On the motivational front, physical exercise has been linked to increased intrinsic motivation and engagement in workplace tasks, as employees often report feeling more focused and energized after brief physical activity interventions (Deci & Ryan, 2008). However, some contrasting findings have emerged, indicating that the effects of physical exercise may be short-lived in specific contexts or that individual differences, such as baseline fitness levels and personality traits, can moderate the outcomes (Bouchard & Rankinen, 2001). While most evidence supports the positive affective benefits of exercise, these nuances suggest that personalized approaches may be necessary to maximize its effectiveness across diverse employee populations.

Emerging evidence highlights the substantial impact of light and color on affective characteristics, with carefully selected light conditions capable of influencing mood, alertness, and overall emotional well-being. Bright, natural light, for example, has been shown to elevate mood, reduce symptoms of depression, and enhance cognitive performance (Viola et al., 2008; Cajochen et al., 2014). Similarly, color tones such as blue or green light can induce calming effects, while red hues may stimulate alertness and arousal (Xie et al., 2022). On another front, sound and music have been extensively studied for their effects on emotions and cognitive functions. Specific tones, rhythms, and musical styles can evoke a range of affective states, from relaxation and calmness to motivation and increased focus, as demonstrated in studies exploring music therapy and its influence on psychological health (Chanda & Levitin, 2013; Koelsch, 2020). Given these findings, a compelling question arises: Could the integration of exercise with light and sound environments, featuring carefully curated colors, tones, and rhythms, further enhance the positive impact on affective characteristics? This integrative approach could potentially amplify mood improvements, reduce anxiety, and boost motivation during exercise sessions. Additionally, tailoring such exercise programs and their accompanying sensory environments to the individual’s current psychophysiological state, including levels of fatigue, stress, and concentration deficits, may maximize their effectiveness and personalization (Sander et al., 2005; Fritz et al., 2013). Another intriguing question is whether this integration of physical exercise with specially designed light and sound environments could produce immediate changes within very short intervals, such as less than 15 min, further supporting the potential for rapid and effective interventions (Basso & Suzuki, 2017). Answers to these questions could open up intriguing possibilities for a holistic approach that merges physical activity with dynamic sensory elements to optimize the emotional well-being of employees and, as a consequence, increase their engagement.

To address these research gaps, we evaluated the effects of an innovative program that integrates physical exercise with specially designed light and sound environments while also being tailored to employees’ current psychophysiological states, including feelings of fatigue, stress, and lack of concentration. The program aimed to examine acute changes in affective characteristics, such as positive and negative emotions, anxiety, and work motivation among employees. Specifically, the aim of this study was to determine whether combining exercise with curated sensory stimuli, selected colors, tones, and rhythms could enhance the beneficial emotional and motivational effects previously attributed to exercise alone. Based on the existing body of research, the following hypotheses were formulated:

**H0:**  *The exercise program with sensory interventions does not result in significant changes of the positive and negative emotions, state anxiety, or work motivation (energy levels and emotional arousal) of employees compared to the control condition.*

**H1:**  *Participating in the exercise program will result in an increase in positive emotions (e.g., enthusiasm, inspiration) and a decrease in negative emotions (e.g., nervousness, irritation), as compared to the control condition.*

**H2:**  *The exercise program will lead to a reduction in state anxiety compared to the control group.*

**H3:**  *Participation in the exercise program will lead to a significant increase in energy levels and emotional arousal, as compared to the control condition.*

The expected results are important because they could provide evidence for an effective, multi-faceted approach to improving employee affect, ultimately resulting in increased well-being and substantial benefits for employers. By fostering a more positive, less anxious, and highly motivated workforce, organizations can capitalize on improved employee well-being, leading to reduced costs related to absenteeism, higher engagement, and a more resilient and productive work environment.

## 2. Materials and Methods

### 2.1. Participants

The study involved 84 participants employed across various sectors (middle age 38.84 ± 10.78, 42.8% males). Selection criteria required participants to be currently employed without restrictions on gender, education level, age, or physical fitness status. Participants were recruited through internal calls within organizations that expressed willingness to participate in the study. All participants were thoroughly informed about the purpose, objectives, and procedures of the study. Prior to testing, each participant signed an informed consent form in accordance with the Helsinki Declaration, affirming their voluntary participation and agreement with all outlined procedures and potential risks. The study received ethical approval from the Ethics Committee of the Faculty of Sport and Physical Education, University of Belgrade (approval number 02-3199/24-2), ensuring compliance with ethical research principles involving human subjects. Special care was taken to ensure that participants did not have any serious medical conditions that could compromise their ability to safely engage in the exercise program.

### 2.2. Instruments

Validated instruments were used to measure affective characteristics, anxiety, and work motivation to assess the impact of the intervention.

#### 2.2.1. Positive and Negative Affect Schedule (PANAS)

The PANAS (Watson et al., 1998) was used to evaluate participants’ immediate emotional state, distinguishing between positive and negative affect. It consists of 20 adjectives describing different feelings, categorized into two subscales: positive affect (e.g., “enthusiastic”, “inspired”) and negative affect (e.g., “nervous”, “upset”). Participants rated their feelings on a five-point Likert scale from 1 (“not at all”) to 5 (“extremely”), with higher scores indicating a stronger presence of the respective affective state.

#### 2.2.2. State-Trait Anxiety Inventory (STAI-S)

State anxiety was measured using the STAI-S (Spielberg et al., 1971), which includes 20 items reflecting varying levels of anxiety, such as “tense” and “calm.” Participants rated their current feelings on a four-point Likert scale from 1 (“not at all”) to 4 (“very much so”), with higher scores indicating increased anxiety.

#### 2.2.3. Motivational State Questionnaire (MSQ-r Short Form)

Work motivation was assessed using a modified version of the MSQ-r short form (Revelle & Anderson, 1999), adapted for the workplace context. This eight-item scale measures two dimensions: energy level (e.g., “I am full of energy”) and emotional arousal (e.g., “I feel relaxed”). Responses were recorded on a five-point Likert scale, with higher scores representing greater motivation. Factor analysis (principal components method with direct oblimin rotation) confirmed the two-factor structure of the instrument.

### 2.3. Study Design

To examine the acute effects of brief exercise programs on the affective characteristics of employees, an experimental study employing a pretest–posttest design was conducted, comprising two groups: an experimental group (*n* = 42) and a control group (*n* = 42). The research was structured into two main sessions, an introductory session and an experimental session, the latter divided into three phases: pretest, intervention, and posttest. The introductory session took place online a few days prior to the experimental session, during which participants completed an anonymous questionnaire using a randomly generated code. This questionnaire collected demographic data such as gender, age, and subjective perception of socio-economic status. It assessed physical activity levels over the previous week via the International Physical Activity Questionnaire (IPAQ) (Craig et al., 2003), job satisfaction using the Job Satisfaction Questionnaire (JSQ) (Spector, 1985), work motivation through the Motivation at Work Scale (MWS) (Gagné et al., 2010), and levels of depression, stress, and anxiety over the previous week using The Depression Anxiety Stress Scale (DASS-21) (Antony et al., 1998). The selection of these specific research tools was guided by their established validity, reliability (Craig et al., 2003; Chen, 2009; Coker et al., 2018; Kim et al., 2022), and relevance to the study aims, all providing insights into factors affecting workplace well-being and performance. Data were used to describe the sample characteristics and to verify the homogeneity between the experimental and control groups, ensuring a balanced comparison for subsequent analyses.

In the experimental session, the pretest phase assessed participants’ baseline affective characteristics using validated tools. The Positive and Negative Affect Schedule (PANAS) evaluated current feelings, the State-Trait Anxiety Inventory (STAI-S) measured anxiety levels, and a modified Motivation for Work Scale (MSQ-r short form) gauged immediate work motivation. During the intervention phase, participants in the experimental group engaged in one of three 14-min exercise programs designed to address specific psychophysiological states such as fatigue, stress, or lack of concentration. Participants selected their preferred program to align with their current state. The exercise took place in a specially designed dark room that incorporated a unique light and sound environment, featuring music and audiovisual effects to enhance the sensory experience and potentially amplify the intervention’s effects. Conversely, the control group watched a neutral animated documentary video to maintain control over external influences. In the posttest phase, participants completed the same set of questionnaires as in the pretest phase, enabling a detailed comparison of changes in affective characteristics. This pretest–posttest design allowed for a comprehensive evaluation of the acute psychological impacts of targeted brief exercise interventions, strengthening the validity of the findings through comparison within and between groups.

### 2.4. Procedures

This study aimed to establish the effectiveness of an intervention program. The dependent variables were represented by data collected through three questionnaires that assessed affective characteristics, while the independent variable was a 14-min exercise program tailored to the participants’ current psychophysiological state, as determined by their subjective feelings of fatigue, stress, or lack of concentration.

The dependent variables included measures of current affective states, anxiety, and work motivation. PANAS was used to assess the participants’ immediate emotional state, capturing both positive and negative affect dimensions, STAI-S was utilized to measure the current state of anxiety, and work motivation was assessed using the MSQ-r short form.

In this study, the independent variable was the presence or absence of an experimental treatment. Participants in the experimental group engaged in one of three 14-min exercise programs specifically tailored to address their current psychophysiological state. Briefly explained to participants, the programs highlighted their intended effects, helping individuals choose the most appropriate option based on whether they felt fatigued, stressed, or lacking concentration. Collectively referred to as the “My Flow” concept, the programs were designed to enhance employees’ affective states and cognitive abilities during work hours. In contrast, the control group watched a 14-min neutral animated documentary video to ensure comparable exposure to less intensive visual and auditory stimuli without physical activity.

**Figure 1:**
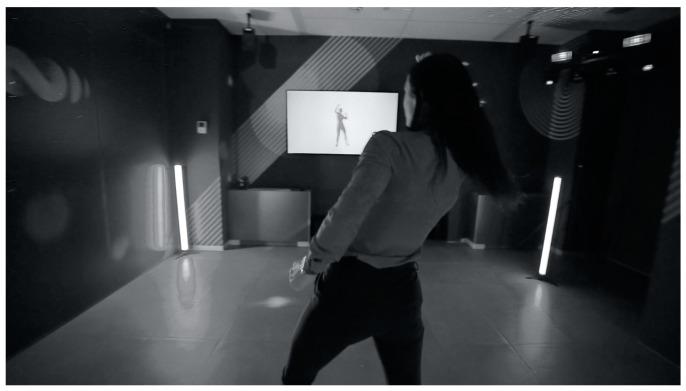
The participant engaged in the ‘Energy’ exercise program within the ‘My Flow’ concept room. The image showcases a female participant performing aerobic-based exercises in a dark room specifically designed to enhance the sensory experience with curated light and sound stimuli.

The ’My Flow’ concept encompassed three distinct programs: ‘Energy’, ‘Vitality’, and ‘Calm’, each targeting different aspects of well-being. Designed by experts in exercise science, these programs incorporated principles of acute exercise physiology and sport psychology to optimize both physical and mental health outcomes for employees of varying fitness levels. Specifically, the ‘Energy’ program focused predominantly on aerobic exercises designed to enhance cardiovascular fitness and boost energy levels. The ‘Vitality’ program emphasized functional strength and mobility exercises to improve physical resilience and prevent injuries, thereby enhancing overall mental state. Finally, the ‘Calm’ program combined flexibility exercises with controlled breathing techniques to promote muscle relaxation and reduce stress. The sessions took place in a specially designed ‘smart room’ equipped to create an immersive light and sound environment tailored to the selected program. The exercise program was led by a human silhouette, prerecorded and projected on the room’s wall, guiding participants through the exercises. Advanced technology adapted the room’s lighting, music, and video content to provide an engaging sensory experience and maximize the benefits of the exercise interventions (for details, see Figure 1). Participants could choose their preferred program based on their current state, promoting inclusivity and accessibility. The exercises were functional, adaptable to individual fitness levels, and required no specialized equipment, making them suitable for diverse workplace settings. Music and audiovisual elements were used strategically to stimulate neurological processes, enhance mood, and boost motivation, with high-quality sound systems delivering an immersive auditory experience. The integration of colored lighting further influenced participants’ psychological states, while visual stimuli, such as nature scenes, contributed to mental relaxation and improved focus.

The control group watched a documentary video (“Black Coffee: The Journey”) (Soulistic Agency, 2017), which was selected for its neutral content and similarity in visual and auditory elements to the “My Flow” programs, albeit at a less intense level. This design aimed to minimize potential confounding effects, ensuring that any observed differences in affective outcomes were attributable to the exercise intervention itself.

### 2.5. Statistical Analysis

Data analysis incorporated both descriptive and inferential statistical methods. Descriptive statistics, including measures of central tendency and dispersion, provided a detailed understanding of data distribution, central values, and variability, forming a basis for further analyses. Inferential analysis primarily involved repeated measures analysis of variance (Repeated Measures ANOVA) to determine if changes in dependent variables were significantly greater in the experimental group compared to the control group. Each ANOVA evaluated the main effects of independent variables and their interactions, with post hoc tests conducted for significant interactions to identify specific differences. Independent variables included time of measurement (pretest and posttest) as a repeated factor and treatment type (experimental and control group) as a non-repeated factor, while dependent variables consisted of scores from measures of affective functioning. Suppose baseline differences between groups were identified (e.g., physical activity level, job satisfaction, work motivation, depression, stress, anxiety), repeated measures analysis of covariance (Repeated Measures ANCOVA) was used, controlling for these variables. The analysis included assumption testing for normality, homogeneity of variances, and sphericity before conducting ANOVA and ANCOVA to assess main effects, interactions, and program impacts. All statistical analyses were performed using the Statistical Package for the Social Sciences (SPSS, Version 25; IBM Corp., Armonk, NY, USA) for accuracy and reliability.

## 3. Results

### 3.1. Preliminary Analysis

Descriptive statistics and an independent samples t-test confirmed that the experimental and control groups were statistically comparable at baseline across all control variables, ensuring the validity of subsequent comparisons (Table 1). 

### 3.2. Main Analysis

**Figure 2:**
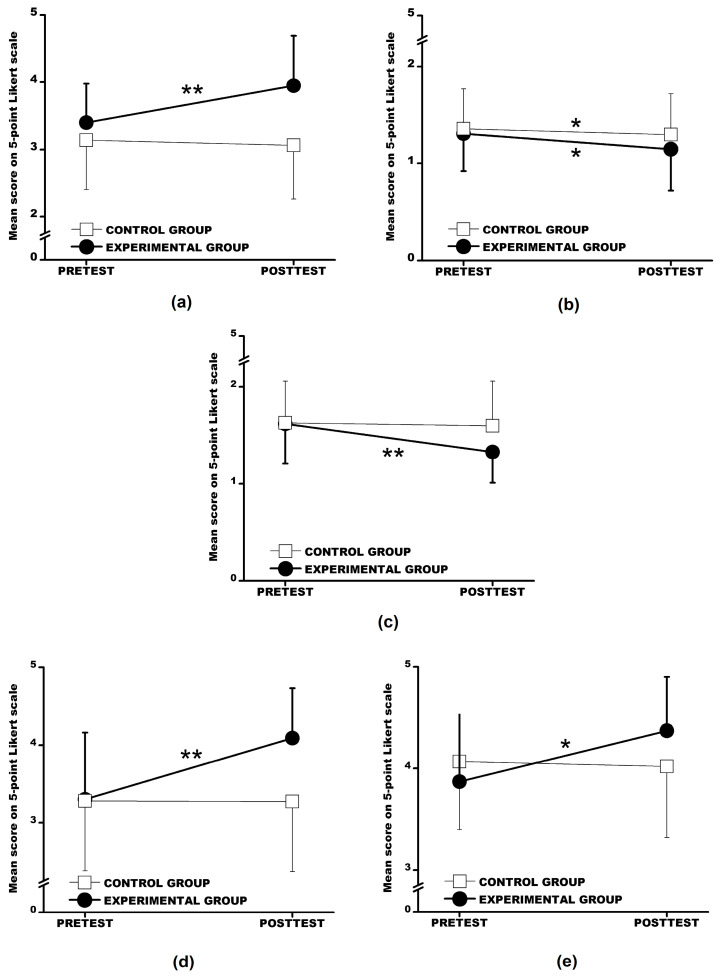
Changes in affective and motivational characteristics from pretest to posttest for the experimental and control groups: (**a**) Positive Emotions (PE_PANAS) ^ψ,β,#^; (**b**) Negative Emotions (NE_PANAS) ^β^; (**c**) State Anxiety (SA_STAI-S) ^β,#^; (**d**) Work Motivation/Energy Level/(WM_El) ^ψ,β,#^; and (**e**) Work Motivation/Emotional Arousal/(WM_Ea) ^ψ,β,#^. Legend: Error bars, Standard Errors; ^ψ^, Significant Main Effect of Group; ^β^, Significant Main Effect of Time; ^#^, Significant Interaction between Group and Time; * *p* < 0.05; and ** *p* < 0.01.

To evaluate the program’s effects, a series of 2 × 2 repeated measures ANOVAs were conducted with group (experimental, control) as a between-subject factor and time (pretest, posttest) as a within-subject factor. Dependent variables included subscales of positive emotions, negative emotions, state anxiety, and work motivation (energy and emotional arousal dimensions). Results are presented in Figure 2.

#### 3.2.1. Positive Emotions (PE_PANAS)

A significant main effect of group, F(1, 82) = 16.35, *p* < 0.01, η^2^ = 0.17, a main effect of time, F(1, 82) = 15.22, *p* < 0.01, η^2^ = 0.16, and a significant interaction between group and time, F(1, 82) = 20.99, *p* < 0.01, η^2^ = 0.20, were observed (Figure 2a). Post hoc tests (Sidak) revealed no significant differences between groups at the pretest, while a significant increase was observed in the experimental group at the posttest (*p* < 0.01). Additionally, within-group comparisons showed significant increases in positive emotions from pretest to posttest for the experimental group (*p* < 0.01), whereas no significant change occurred in the control group.

#### 3.2.2. Negative Emotions (NE_PANAS)

A main effect of time was significant, F(1, 82) = 5.01, *p* < 0.05, η^2^ = 0.06, indicating an overall reduction in negative emotions from pretest to posttest across both groups (Figure 2b). However, no significant main effects for group or interaction effects were observed. Post hoc comparisons (Sidak) confirmed that both groups experienced significant reductions in negative emotions over time (*p* < 0.05).

#### 3.2.3. State Anxiety (SA_STAI-S)

Significant main effects of time, F(1, 82) = 17.87, *p* < 0.01, η^2^ = 0.18, and interaction effects, F(1, 82) = 11.37, *p* < 0.01, η^2^ = 0.12, were found (Figure 2c). Post hoc tests (Sidak) revealed no significant differences between groups at the pretest, while significant reductions in anxiety were observed at the posttest for the experimental group compared to the control group (*p* < 0.01). Furthermore, within-group analyses indicated that anxiety levels significantly decreased from pretest to posttest in the experimental group (*p* < 0.01), whereas no significant change occurred in the control group.

#### 3.2.4. Work Motivation (Energy Level, WM_El)

For the energy dimension of work motivation, a significant main effect of group, F(1, 82) = 6.15, *p* < 0.05, η^2^ = 0.07, time, F(1, 82) = 44.71, *p* < 0.01, η^2^ = 0.35, and a significant interaction effect, F(1, 82) = 48.36, *p* < 0.01, η^2^ = 0.36, were observed (Figure 2d). Post hoc analyses (Sidak) indicated no significant pretest differences between groups, but a significant increase in work motivation was noted for the experimental group at posttest (*p* < 0.01). Within-group analyses showed a significant improvement in energy level of work motivation from pretest to posttest for the experimental group (*p* < 0.01), while no significant change occurred for the control group.

#### 3.2.5. Work Motivation (Emotional Arousal, WM_Ea)

Main effects of time, F(1, 82) = 8.94, *p* < 0.01, η^2^ = 0.10, and interaction effects, F(1, 82) = 12.29, *p* < 0.01, η^2^ = 0.13, were identified (Figure 2e). Post hoc tests (Sidak) revealed no significant differences between groups at the pretest, but significant improvements were noted at the posttest for the experimental group (*p* < 0.05). Additionally, within-group comparisons demonstrated a significant increase in emotional arousal of work motivation for the experimental group from pretest to posttest (*p* < 0.05), with no significant changes in the control group.

The mean scores on a 5-point Likert scale, as presented in Figure 2, offer valuable insights into the impact of the intervention on positive emotions, negative emotions, state anxiety, and work motivation. The experimental group exhibited a significant increase (*p* < 0.01) in positive emotions (PE_PANAS), rising from M = 3.40 (pretest) to M = 3.95 (posttest), whereas the control group showed no improvement (pretest: M = 3.14; posttest: M = 3.06) (Figure 2a). A significant decrease (*p* < 0.05) in negative emotions (NE_PANAS) was observed in the experimental group (pretest: M = 1.31; posttest: M = 1.15), while the control group exhibited minimal change (pretest: M = 1.36; posttest: M = 1.30) (Figure 2b). The experimental group also experienced a significant reduction (*p* < 0.01) in state anxiety (SA_STAI-S), dropping from M = 1.62 (pretest) to M = 1.33 (posttest), whereas the control group showed no meaningful change (pretest: M = 1.63; posttest: M = 1.60) (Figure 2c). A notable increase (*p* < 0.01) in energy-related work motivation (WM_El) was observed in the experimental group, rising from M = 3.30 (pretest) to M = 4.09 (posttest), while the control group remained stable (pretest: M = 3.28; posttest: M = 3.27) (Figure 2d). Both groups demonstrated relatively high baseline emotional arousal (WM_Ea) related to work motivation. However, the experimental group exhibited a significant increase (*p* < 0.05) from M = 3.87 (pretest) to M = 4.37 (posttest), while the control group experienced a slight decline (pretest: M = 4.07; posttest: M = 4.02) (Figure 2e).

The findings highlight the efficacy of the sensory-enriched exercise intervention in improving positive emotions, reducing anxiety, and enhancing both energy and emotional arousal dimensions of work motivation. Limited effects were observed for negative emotions, which decreased over time across both groups without significant group differences. Detailed results are summarized in Table 1 and illustrated in Figure 2a–e. These results underscore the potential of integrating physical activity with sensory-rich environments to enhance employees’ emotional well-being and work motivation within short intervention intervals.

## 4. Discussion

The aim of this study was to examine the immediate effects of an innovative exercise program, known as the ‘My Flow’ concept, conducted in a sensory-rich environment on employees’ affective characteristics. The results showed significant improvements in the experimental group, supporting the hypothesis that brief, tailored physical activity enhances emotional states and work motivation. Positive affect significantly increased, while anxiety levels decreased, indicating the program’s effectiveness in boosting enthusiasm and reducing stress. Although the reduction in negative affect was greater in the experimental group, the difference was not statistically significant, suggesting variability in individual responses. Both tested dimensions of work motivation also showed a significant increase, underscoring the program’s potential to enhance workplace engagement. These findings highlight the utility of short, adaptive exercise programs within sensory environments to improve employees’ emotional well-being.

The results of this study align with the growing recognition of the critical role of those affective characteristics, such as positive and negative emotions, motivation, and overall workplace well-being (Fredrickson, 2013; Bakker & Demerouti, 2017). Positive emotions, for instance, have been consistently associated with enhanced creativity, collaboration, and job satisfaction, whereas negative emotions related to stress and frustration are linked to diminished focus and increased error rates (Fisher, 2010; Schaufeli, 2015). Similarly, anxiety has been identified as a key barrier to workplace efficiency, impairing decision-making and cognitive flexibility (Lepine et al., 2005). Given the substantial costs associated with workplace stress and disengagement, interventions aimed at improving emotional states and motivation are increasingly viewed as essential components of organizational strategies (Deci & Ryan, 2000; Cropanzano et al., 2017). The findings of this study not only reinforce these established associations but also emphasize the potential of brief, targeted physical activity interventions in sensory-rich environments to address these critical challenges.

The integration of exercise with curated light and sound environments significantly enhances its positive impact on affective characteristics. This study demonstrated substantial improvements in positive affect and reductions in anxiety among participants exposed to such multi-sensory interventions, highlighting the additive effects of sensory elements. Research shows that blue and green light tones promote calmness, while red hues enhance alertness, complementing the psychological benefits of exercise (Chellappa et al., 2011; Xie et al., 2022). Similarly, rhythmic music engages the brain’s reward system, boosting mood and motivation (Chanda & Levitin, 2013; Koelsch, 2020). Immersive sensory environments leveraging coherence and novelty further stimulate emotional well-being and cognitive outcomes (Chellappa et al., 2014), while recent studies demonstrate the role of multi-sensory environments in reducing mental fatigue and improving task engagement (Chang et al., 2012). This synergy likely enhanced participant immersion and mindfulness, amplifying the psychological effects of exercise. The findings align with the broaden-and-build framework, which posits that positive emotions expand cognitive and behavioral capacities (Fredrickson, 2013). These results underline the potential of sensory-enriched exercise as an innovative workplace intervention to enhance employee well-being, warranting further research to refine these approaches for diverse populations.

Tailoring exercise programs and sensory environments to individuals’ psychophysiological states is strongly supported by neuroscience and psychology, offering insights into how such personalization enhances effectiveness. This study’s findings align with neuroscience research, showing that exercise stimulates brain regions like the prefrontal cortex, enhancing emotional resilience and reducing stress through neuroplastic changes (Ekkekakis, 2017). Complementary sensory inputs, such as music or light tailored to mood, modulate neural activity, engage reward circuits, and lower stress-related cortisol levels (Chellappa et al., 2011; Koelsch, 2020). Psychology studies further demonstrate that interventions aligned with individual mood states, such as rhythmic music or calming light, can enhance cognitive focus and emotional regulation by reducing fatigue and restoring mental resources (Xie et al., 2022). By addressing immediate states like fatigue or stress, these tailored approaches maximize engagement and effectiveness, leveraging the brain’s adaptive mechanisms while fostering emotional balance. These findings underscore the potential of neuroscience-informed, personalized interventions in workplace wellness programs, offering scalable strategies to improve employee well-being.

The integration of physical exercise with specially designed light and sound environments demonstrates significant potential for producing immediate changes in affective states within short intervals, such as under 15 min. Research in kinesiology confirms that even brief exercise sessions can enhance mood, reduce anxiety, and boost motivation through mechanisms like increased endorphin release and improved cardiovascular function (Loprinzi et al., 2018). When paired with sensory inputs such as dynamic lighting and rhythmic music, these benefits are further magnified by stimulating sensory pathways that regulate mood and cognitive focus (Tarr et al., 2014). Moderate-intensity exercise is particularly effective, delivering rapid psychological benefits without inducing fatigue associated with higher intensities (Chang et al., 2012). In this study, the 14-min multi-sensory exercise intervention significantly improved positive affect and motivation, underscoring the efficacy of short, structured physical activity in enhancing emotional well-being. These findings highlight the potential for such interventions to be implemented in workplace settings as a rapid, scalable solution to improve employee mental health.

Building on the insights gained from the immediate effects of the “My Flow” concept, it is important to acknowledge both the limitations and strengths of the present study. A notable limitation is the inability to investigate the specific effects of the three tailored exercise programs—“Energy”, “Vitality”, and “Calm”—on affective characteristics, as the experimental group was divided into smaller subgroups, reducing statistical power. While additional analyses revealed no significant differences among the programs, these findings should be interpreted with caution due to the limited sample size. Nonetheless, the fundamental premise of the “My Flow” concept—to adapt to employees’ current psychophysiological states and reset affective characteristics to an equally optimal level—aligns with the observed outcomes, underscoring the effectiveness of the intervention as a whole. Despite these limitations, the study’s innovative approach, combining physical exercise with sensory elements, offers significant strengths. The curated light and sound environments enhanced the psychological benefits of exercise, making the intervention practical and scalable for workplace applications. Furthermore, the personalized design, which allowed participants to select a program aligned with their immediate needs, fostered greater engagement and adherence. Future research should delve deeper into the longitudinal effects of these interventions, examining their sustained impact on workplace emotional resilience and mental health. Investigations could explore the interplay between sensory elements and diverse exercise modalities over extended periods, assessing their combined influence on cognitive and affective outcomes. Additionally, neurophysiological studies could provide critical insights into mechanisms such as changes in brain activity patterns and stress hormone levels, advancing our understanding of how these interventions operate at both psychological and biological levels.

## 5. Conclusions

This study underscores the transformative potential of integrating brief, tailored exercise programs with sensory-rich environments to enhance employees’ affective characteristics and work motivation. The findings reveal that even short interventions can significantly improve emotional states and reduce anxiety, demonstrating the feasibility and effectiveness of such approaches in workplace settings. These results validate the “My Flow” concept as a promising model for promoting workplace well-being. Practical applications of these findings are vast and innovative. Organizations could integrate sensory-enriched exercise spaces, akin to “smart rooms”, into office environments, allowing employees to access tailored physical activity programs during breaks. Mobile applications could extend these interventions to remote and hybrid work settings, using artificial intelligence (AI) to analyze users’ current states and recommend personalized programs. Moreover, partnerships between employers and health-tech companies could facilitate wearable devices that adapt light, sound, and exercise intensity in real time, ensuring a seamless and immersive experience. For broader public health applications, such interventions could be adapted for high-stress professions, such as healthcare or emergency services, providing rapid emotional recalibration during demanding work schedules. These strategies highl ight the practical potential of leveraging the principles of personalized, multi-sensory exercise to optimize not only workplace engagement but also overall quality of life.

## Figures and Tables

**Table 1:** Variables assessed included age, socio-economic status (SES), physical activity (PA), job satisfaction (JS), dimensions of work motivation, and levels of depression, anxiety, and stress. No significant baseline differences were identified between groups.

	Experimental Group					Control Group				
	*n*	Min	Max	Mean	SD	*n*	Min	Max	Mean	SD
Age	42	23.00	60.00	39.65	10.12	42	20.00	65.00	38.05	11.45
SES	42	35.00	90.00	67.67	13.34	42	30.00	98.00	69.75	15.41
PA	42	99.00	3828.00	1601.00	866.87	42	33.00	4906.00	1701.00	1200.09
JS	42	2.56	5.81	4.13	0.73	42	2.58	5.69	4.09	0.82
WM_In	42	2.33	5.00	3.86	0.81	42	2.00	5.00	3.73	0.91
WM_Id	42	1.33	5.00	3.43	0.97	42	1.33	5.00	3.58	0.98
WM_Int	42	1.00	5.00	2.76	1.05	42	1.00	4.67	2.80	1.10
WM_Ex	42	1.33	5.00	3.09	0.94	42	1.00	4.67	3.47	0.79
D	42	1.00	3.71	1.58	0.62	42	1.00	3.14	1.49	0.61
A	42	1.00	3.43	1.49	0.57	42	1.00	3.29	1.45	0.58
S	42	1.00	3.57	2.17	0.71	42	1.00	3.86	2.07	0.81

Legend: *n*, Number of Participants; SES, Socio-Economic Status; PA, Physical Activity; JS, Job Satisfaction; WM_In, Intrinsic Motivation for Work; WM_Id, Identified Motivation for Work; WM_Int, Introjected Motivation for Work; WM_Ex, Extrinsic Motivation for Work; D, Depression; A, Anxiety; and S, Stress.

## Data Availability

Data and materials are available from the corresponding author upon reasonable and appropriate request.
